# Mean core to peripheral temperature difference and mean lactate levels in first 6 hours of hospitalisation as two indicators of prognosis: an observational cohort study

**DOI:** 10.1186/s12887-020-02418-w

**Published:** 2020-11-10

**Authors:** Aashish Gupta, Jacob Puliyel, Bhawana Garg, Pramod Upadhyay

**Affiliations:** 1 Department of Paediatrics, St. Stephens Hospital, Tis Hazari, Delhi, India; 2 Independent Statistician, 135 Bhagirathi, Sector 9, Rohini, Delhi, India; 3 National Institute of Immunology, New Delhi, India

**Keywords:** Core to peripheral temperature difference, Pediatric risk of mortality (PRISM II), Lactate clearance, Mean lactate

## Abstract

**Background:**

To study mean core to peripheral temperature difference (CPTD) and the mean lactate levels over the first 6 h of admission to hospital, as indicators of prognosis in critically ill children.

**Methods:**

A prospective observational study in a tertiary level Pediatrics ICU in Delhi, India. Seventy eight paediatric patients from 1 month to 12 years were studied. Children with physical trauma, post-surgical patients and patients with peripheral vascular disease were excluded. Core temperature (skin over temporal artery) to peripheral temperature (big toe) difference was measured repeatedly every minute over 6 h and mean of temperature difference was calculated. Pediatric Risk of Mortality (PRISM) II, lactate clearance and mean lactate levels during that time were also studied. In-hospital mortality was used as the outcome measure.

**Results:**

*Mean temperature difference*

During the first 6 h after admission the mean temperature difference was 9.37 ± 2 °C in those who died and 3.71 ± 2.27 °C in those who survived (*p* < 0.0001). The area under the receiver operating curve (AUROC) was 0.953 (*p* < 0.0001). The comparable AUROC of PRISM II was 0.999 (*p* < 0.0001).

*Mean Lactate*

Mean lactate level in the first 6 h was 7.1 ± 2.02 mg/dl in those who died compared to 2.86 ± 0.87 mg/dl in those who survived (*p* < 0.0001). The AUROC curve for mean lactate was 0.989 (95% CI = 0.933 to 0.999; *p* < 0.0001). AUROC for the lactate clearance was 0.682 (*p* = 0.0214).

**Conclusions:**

The mean core to peripheral temperature difference over the first 6 h is an easy-to-use and non-invasive method that is useful to predict mortality in children admitted to the Pediatric ICU. The mean lactate during the first 6 h of Pediatric ICU admission is a better index of prognosis than the lactate clearance over the same time period. They may be used as components of a scoring system to predict mortality.

**Supplementary Information:**

The online version contains supplementary material available at 10.1186/s12887-020-02418-w.

## Background

The task force on circulatory shock has stressed the importance of assessing tissue perfusion [1]. Skin temperature can be used as an index of perfusion and it is influenced by the cardiac output and the tone of the arteriolar vessels [2]. In seriously ill patients, microcirculatory derangements that result from impaired perfusion has been used to predict survival [3]. Schey and colleagues have used the core to peripheral temperature difference as a marker of perfusion and hemodynamic stability in critically ill patients [4]. Peripheral skin temperature has been found to be a better predictor of in-hospital mortality than the mean arterial blood pressure [5–7]. The core to peripheral temperature difference has also been validated in paediatric patients as a predictor of mortality [2, 8, 9]*.*

The response to resuscitation and the normalisation of core to peripheral temperature difference during hospitalisation has also been studied as an index of prognosis. Examining the time to normalisation of the core to peripheral difference, Herandez has shown that if the central to toe temperature difference normalises within 6 h, it was predictive of the normalisation of serum lactate levels within 24 h [10]. Joly et al. found that, 3 h after admission, if the toe temperature is less than 27 °C, the likelihood of death was high [11]. In a retrospective study in adults admitted to the intensive care unit, Houwink and colleagues found that the arithmetic mean of the core to peripheral temperature difference (delta T), measured intermittently over the first 24 h of admission, was an independent predictor of in-hospital mortality [12]. On the other hand, they found that the core-to-peripheral temperature difference at admission was not related to mortality.

In 2015 Cuesta-Frau and colleagues developed a novel device capable of monitoring and recording the core to peripheral temperature difference every 30 s [13]. We postulate that high frequency recording of temperature and temperature difference as described by Cuesta-Frau (referred to as ‘continuous recording of temperature’ in this article) may be more useful in predicting mortality than the intermittent readings utilised by Houwink and colleagues [12] as it affords much greater granularity of data. The mean of the numerous readings of the core to peripheral temperature difference collected over the first 6 h may help identify patients who normalise early, compared to those that normalise slowly. We hypothesize that continuous monitoring over the first 6 h of admission may be used to predict mortality in children admitted to the paediatric intensive care unit (PICU). It has the distinct advantage of being non-invasive and it is not dependent on blood test reports. To the best of our knowledge this has not been studied previously.

Another parameter we investigated was the excessive production and poor clearance of lactate which are responsible for the high blood lactate levels in patients with circulatory failure. Studies have shown that the serum lactate level at hospital admission is a biomarker of prognosis [14–18].

In adult patients with severe sepsis, Nguyen and colleagues have demonstrated that ‘lactate clearance’ during the first 6 h of hospital admission (defined as the percentage decrease in lactate [(lactate_initial_ – lactate_after 6 h_) / lactate_initial_ × 100] was a better index of prognosis than lactate levels at admission [19]. We have previously demonstrated the usefulness of lactate clearance to predict in all-cause mortality among children admitted to a PICU [20].

Most studies to-date have focused on lactate clearance in patients who had high lactates at admission. On the other hand, patients who have normal lactate levels initially, have little lactate to clear. These patients have good prognosis even though they show up as having poor clearance (no reduction in lactate values) using the lactate clearance formula. This has been noted by Jones who reviewed lactate clearance and found that it has no utility in up to 30% of patients with septic shock who have lactate levels < 2 mmol/L [21]. We hypothesized that the mean lactate over the initial 6 h of hospital admission [(lactate_initial_ + lactate_after 6 h_) / 2] would be a more reliable indicator of prognosis applicable consistently to all patients, regardless of the initial lactate levels - than lactate clearance over 6 h (which is reliable only in patients with high initial lactate levels). To the best of our knowledge, this has not been investigated previously.

Put together, we studied the prediction of mortality using continuously recorded core to peripheral temperature difference (CPTD), against other indicators of prognosis, namely PRISM score (acquired over the first 24 h of admission) [22] and lactate clearance during the first 6 h of hospital admission (defined as the percentage decrease in lactate [(lactate_initial_ – lactate_after 6 h_) / lactate_initial_ × 100 [15]. We also studied the prediction of mortality using the mean lactate levels over 6 h, to examine if it performed better lactate clearance.

## Methods

The study was approved on 22 August 2016 by the Institutional Ethics Committee of St Stephens Hospital, Delhi, India. We conducted a prospective observational study in a 6-bedded pediatric intensive care unit (PICU) in a tertiary hospital in Delhi, India.

We included patients older than 1 month and less than 12 years of age whose clinical condition necessitated admission to the PICU, subject to obtaining written consent for study participation from parents. We excluded children with physical trauma, post-surgical patients and patients with peripheral vascular disease.

We used a temperature sensor LM35 (Texas Instruments, USA) mounted on a stainless-steel disk of 8 mm diameter and 0.5 mm thickness for reading temperature. This sensor gives an output of 10 mV/°C and 0.00 V at 0 °C. The output was fed to an analog-to-digital converter and the digital data and time were logged on a microSD card. The sampling frequency was 1/ s and the output was recorded as the mean of 60 readings, every minute.

Central temperature was measured with a probe placed over the temporal artery on the parietal bone as described by Vinkers, et al. [23]. A probe on the volar surface of the big toe, measured the peripheral temperature, following the method described by Hernandez et al. [10].

The temperature difference over the first 6 h of admission was measured every minute and the mean of all these readings was the parameter we were primarily interested in evaluating. PRISM II score [22] was calculated as also the lactate clearance [15] and the mean lactate over 6 h of admission [17, 20]. Mortality was evaluated against each of these parameters.

### Sample size calculation

Houwink et al. [12] found the mean core to peripheral temperatures difference (delta T) was 4.6 in those with good prognosis and it was 5.9 in those who died. Standard deviation in both groups was 1.5. Assuming similar differences in our sample and a mortality of 20% among PICU admissions; for 80% power and an alpha error of 0.05, we calculated we would need a sample of 78 patients.

### Statistical analysis

Data was entered in MS Excel spreadsheet and analysis was done using Statistical Package for Social Sciences (SPSS) version 21.0.

The primary outcome of interest was mortality against the mean core to peripheral temperature difference. We also examined mortality against PRISM II, lactate clearance and mean lactate values. Normality of data was tested by the Kolmogorov-Smirnov test. If normality was rejected, then a non-parametric test was used. Quantitative variables were compared using Independent T test/Mann-Whitney Test (when the data sets were not normally distributed) between the two groups. Odds ratio with 95% CI was calculated to find out the risk of mortality. Spearman rank correlation coefficient was used to assess the association of various non-parametric parameters with each other. Receiver operating characteristic (ROC) curve was used to find the cut-off point of parameters for predicting mortality and comparison of AUC was performed, to find the best predictor of mortality. A *p*-value of < 0.05 was considered statistically significant.

## Results

**Fig. 1:**
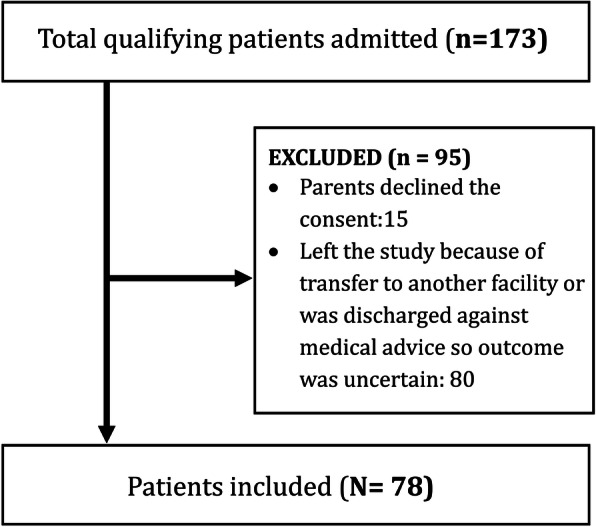
Flow diagram of eligible patients included in study

Data from 78 children enrolled was collected by April 2018. Fifteen eligible children could not be included in the study because their parent did not consent to participate (Fig. 1).

The mean age of the group studied was 2.87 years (± 3.19) (Table 1). The highest representation was from children below = < 6 months [*N* = 20 (25.64%)]. Thirty nine patients were admitted for respiratory diseases, 10 children with gastrointestinal diseases, 19 patients with shock, 16 patients had CNS diseases, 5 children had dengue and 4 children had liver disorders. Some patients had involvement of more than 1 organ system. The study population was analysed in two groups, according to whether they died or survived.

**Table 1:** Summary of the study samples

	Survived	Dead	Remarks
	*N* = 62 79.49%	*N* = 16 20.51%	Total *N* = 78
Mean Age (Years)	2.36 ± 2.89	4.76 ± 3.69	Overall: 2.87 ± 3.19
Male	*N* = 51 83.61%	*N* = 10 16.39%	*N* = 61
Female	*N* = 11 64.71%	N = 6 35.29%	*N* = 17
Core to Peripheral Temperature Difference (CPTD)	Mean ± SD: 3.71 ± 2.27 Range: 0.2–9.9 Median: 3.04 IQR: 2.157–4.970	Mean ± SD: 9.37 ± 2.00 Range: 3.65–11.98 Median: 9.65 IQR: 9.010–10.755	AUROC: 0.953 (*p* < 0.0001) 95% CI: 0.879–0.988
PRISM± SD	5.34 ± 2.55	24.12 ± 8.92	AUROC: 0.999 (*p* < 0.0001) 95% CI: 0.951–1.00
Mean Lactate mg/dl ± SD	2.86 ± 0.87	7.1 ± 2.02	AUROC: 0.989 (*p* < 0.0001) 95% CI: 0.933–0.999
Lactate Clearance mg/dl ± SD	21 ± 25	−5.6 ± 47.2	AUROC: 0.682 (*p* = 0.021) 95% CI: 0.567–0.783
Lactate level (mg/dl) 0 to 2	6	0	
Lactate level (mg/dl) 2.1 to 3	23	1	
Lactate level (mg/dl) 3.1 to 5	26	3	
Lactate level (mg/dl) 5.1 to 10	7	8	
Lactate level (mg/dl) > 10	0	4	

### Mean CPTD over 6 h

**Fig. 2:**
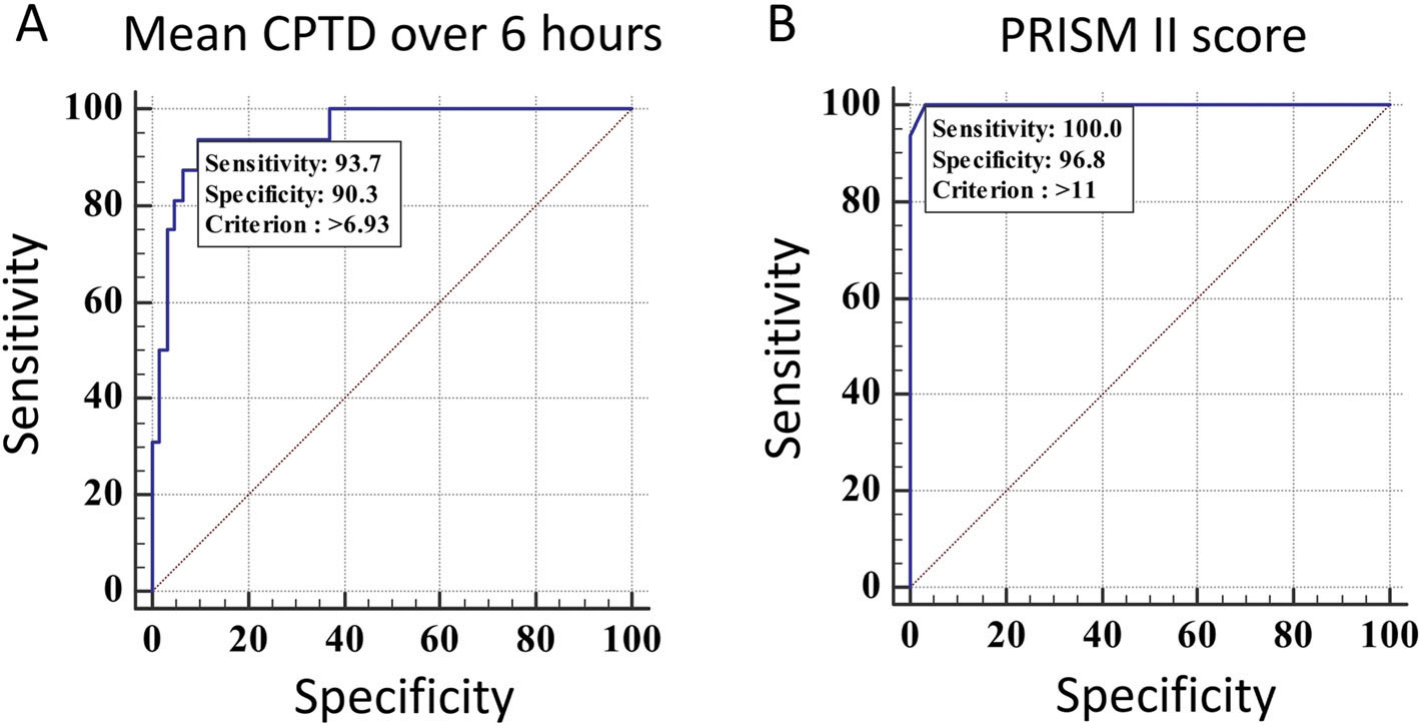
AUROC for the mean core to peripheral temperature difference (CPTD) (Panel **a**) and for PRISM (Panel **b**)

The mean CPTD in the two groups according to survival is shown in the Table 1. During the first 6 h after admission the mean difference was 9.37 ± 2 °C in those who died and 3.71 ± 2.27 °C in those who survived (*p* < 0.0001) (Table 1). The area under the receiver operating curve (AUROC) was 0.953 [95% CI: 0.879 to 0.988] (Fig. 2a). At a cut off of > 6.93, the odds of death were 140.8 times above the cut-off [(*p* < 0.0001); 95%CI:15.87 to 1265.8].

### PRISM II score

AUROC for PRISM II was 0.999. At a cut off > 11, the odds of death were 833.3 times higher [(*p* < 0.0001); 95% CI:36.52 to 17,543.34] (Fig. 2b).

### Lactate levels

The lactate levels at admission are shown in the Table 1. Six of the children studied had normal lactate levels at admission (< 2 mg/dl) and 30 had levels below 3 mg/dl.

The lactate levels at admission, at 6 h, lactate clearance and the mean lactate levels over the first 6 h, were significantly higher among those who died compared to survivors. Mean lactate level in the first 6 h in those who died was 7.1 ± 2.02 mg/dl as compared to 2.86 ± 0.87 mg/dl in those who survived (*p* < 0.0001). The mean lactate clearance was 15.3 ± 32.5% in the sample as a whole. The mean lactate clearance of the children who died was − 5.6 ± 47.2% compared to 21 ± 25% in children who survived (*p* = 0.025).

**Fig. 3:**
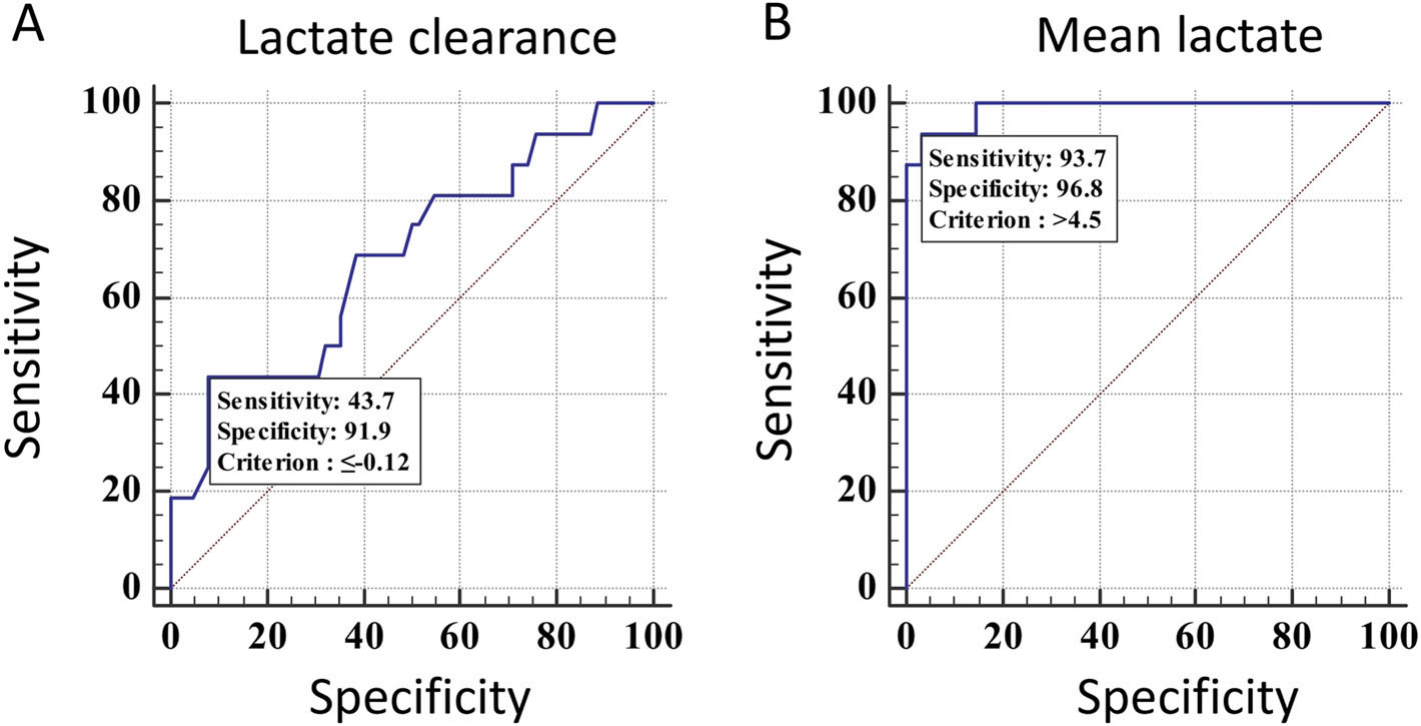
AUROC for lactate clearance (**a**) and for mean lactate over 6 h (**b**)

Figure 3a is ROC curve for mortality against lactate clearance. AUROC for this was 0.682 (*p* = 0.02; 95% CI = 0.57 to 0.78). At a lactate clearance cut-off of < − 12%, the odds of death were 8.87 times higher above than below the cut off [*p* = 0.002; 95% CI: 2.31 to 34.06].

Lactate clearance was not significantly correlated with PRISM II score. (Spearman’s coefficient of rank correlation of 0.0129 [95% CI: − 0.210 to 0.24; *p* = 0.91).

Figure 3b is ROC curve for mortality against mean lactate levels over 6 h. AUROC was 0.989 (95% CI: 0.933 to 0.999; *p* < 0.0001). At a cut-off of > 4.5 the odds of death were 450 times higher above the cut-off compared to below it [*p* < 0.0001; 95% CI: 38.21 to 5300.34].

Mean lactate over 6 h showed a strong correlation with PRISM II score with the Pearson correlation coefficient (r) of 0.689 (*p* < 0.0001).

## Discussion

We found it useful to monitor core to peripheral temperature difference continuously. A high mean CPTD over the first 6 h of hospitalisation was significantly associated with mortality. AUROC curve was 0.953. If the mean difference was > 6.93, the odds of death were 140.8 times higher. Children who respond quickly to treatment and normalise their core to peripheral temperature-difference (and so have a low mean core to peripheral difference) had a better prognosis.

Monetary constraints prompted us to utilise PRISM II as this was available free to use in the public domain rather than PRISM III. We found that PRISM II was a strong predictor of mortality in this study sample. Using PRISM II, the AUROC was better (0.999) than the AUROC for mean CPTD (0.953), but only marginally. Temperature recordings are measured non-invasively, unlike PRISM scoring, which requires blood testing. We anticipate that the prognosis prediction would be better using PRISM III than it is with PRISM II but the scope for improvement in our sample is likely to be marginal, given the AUROC of 0.999 with PRISM II.

### Lactate values

Our study found that mean lactate performed nearly as well as PRISM II in mortality prediction whereas lactate clearance was not such a good predictor. In this study lactate clearance showed little correlation to PRISM II or mortality. Six of the patients in the study had lactate levels below 2 mg/dl at admission and 30 had levels below 3 mg/dl (Table 1). These patients had good prognosis, although by the definition used for the study, they had low lactate clearance. Jones has previously reported that lactate clearance has no utility in up to 30% of patients with septic shock who have lactate levels < 2 mmol/L [21]. Employing mean lactate values allows patients with normal lactates to be included in the evaluation of prognosis.

### Limitations

Some serious limitations need to be borne in mind when interpreting the results of our study and those of others who use the core to peripheral difference to prognosticate. These finding probably apply only to patients in cold shock. In cold shock there is decreased cardiac output and increased systemic vascular resistance. In warm shock, on the other hand, there is increased cardiac output and decreased systemic vascular resistance. Warm shock often manifests as fluid-resistant shock with poor prognosis [24]. In such cases of hyperdynamic shock with vasodilatation, the CPTD may not accurately predict prognosis.

There are factors that cause a rise in lactate levels that are unrelated to the severity of illness. Resuscitation with Ringer’s lactate [25] or large volume blood transfusions can raise the lactate levels. Use of vasopressors in resuscitation may also cause a rise in lactate levels [26]. Further studies are needed to see how these factors influence mortality predictions using the mean lactate values and how one can compensate for these factors when prognosticating.

This is a single centre study and has the attendant limitations regarding generalisability of the findings to other centres catering to children with a different spectrum of disease and severity of illness. Further multi-centre studies would be valuable for validation of our findings. Investigations need to be done to see how best to identify and exclude patients with warm shock when evaluating prognosis using this method.

## Conclusions

1. The mean CPTD over the first 6 h was is an easy to use and non-invasive method useful to predict mortality in children admitted to the PICU.

2. The mean lactate during the first 6 h of PICU admission is a better index of prognosis than the lactate clearance over the same time period.

These may be used as components of a scoring system for prognosis prediction.

## Supplementary Information

**Additional file 1.** Individual patient’s data.

## Data Availability

All data generated or analyzed during this study are included in this published article and its supplementary information files.

## Abbreviations

AUROC: Area Under the Receiver Operating Curve
CPTD: Core to Peripheral Temperature Difference
PICU: Pediatric Intensive Care Unit
PRISM: Pediatric Risk of Mortality
SD: Standard Deviation
